# Differential HDAC1 and 2 Recruitment by Members of the MIER Family

**DOI:** 10.1371/journal.pone.0169338

**Published:** 2017-01-03

**Authors:** Roya Derwish, Gary D. Paterno, Laura L. Gillespie

**Affiliations:** Terry Fox Cancer Research Laboratories, Division of BioMedical Sciences, Faculty of Medicine, Memorial University of Newfoundland, St. John’s, Newfoundland and Labrador, Canada; Medical College of Wisconsin, UNITED STATES

## Abstract

The *mier* family consists of three related genes encoding ELM2-SANT containing proteins. MIER1 has been well characterized and is known to function in transcriptional repression through its ability to recruit HDAC1 and 2. Little is known about MIER2 or MIER3 function and no study characterizing these two proteins has been published. In this report, we investigate MIER2 and MIER3 localization and function. Confocal analysis revealed that, while MIER2 and MIER3 are mainly nuclear proteins, a substantial proportion (32%) of MIER2 is localized in the cytoplasm. Co-immunoprecipitation experiments demonstrated that the MIER proteins do not dimerize; that MIER2, but not MIER3, can recruit HDACs; and that recruitment is cell line-dependent. MIER2 was associated with HDAC1 and HDAC2 in HEK293 cells, but only with HDAC1 in MCF7 and HeLa cells. Little or no MIER3 co-immunoprecipitated with either HDAC1 or 2 in any of the three cell lines tested. By contrast, HDAC1 and 2 were readily detected in MIER1α complexes in all three cell lines. Histone deacetylase assays confirmed that MIER2, but not MIER3 complexes, have associated deacetylase activity, leading to the conclusion that MIER3 does not function in HDAC recruitment in these cell lines. In contrast to what has been reported for other ELM2-SANT associated HDACs, addition of D-myo-inositol-1,4,5,6-tetrakisphosphate led to only a small increase in MIER1α associated deacetylase activity and no effect on that associated with MIER2. Deletion analysis revealed that HDAC recruitment occurs through the ELM2 domain. Finally, using site-directed mutagenesis, we show that, like MIER1, ^228^W in the ELM2 domain is a critical residue for HDAC recruitment by MIER2.

## Introduction

The *mier* family consists of three genes encoding related proteins with conserved primary sequence, particularly in the ELM2 and SANT domains. *Mier1* is the prototypical member and was identified as a fibroblast growth factor early response gene activated during mesoderm differentiation in *Xenopus* [1]. Several MIER1 isoforms have been characterized [2]; each isoform contains central ELM2 and SANT domains with divergent N- & C- termini. There are two C-terminal isoforms, α and β, as well as two N-terminal isoforms that result from alternate inclusion of an exon encoding a nuclear export signal (NES; 3A isoform) [3].

Both MIER1α and MIER1β have been shown to function as transcriptional repressors through their ability to recruit HDAC1/2 activity [4] and to interfere with Sp1 promoter binding [5]. Additional studies focused on the α isoform of MIER1, showing that it interacts with estrogen receptor α (ERα) and that stable expression of MIER1α under the control of the Tre promoter in T47D breast carcinoma cells inhibited estrogen-stimulated colony growth [6]. Immunohistochemical examination of breast tumour samples revealed a shift in the subcellular localization of MIER1α, from the nucleus to the cytoplasm, during breast cancer progression [6]. While the mechanism responsible for shuttling MIER1α out of the nucleus in breast cancer patients is not known, alternative splicing to include the NES [3] or treatment of breast carcinoma cells with insulin or insulin-like growth factor 1 (IGF1) [7] were both shown to produce a similar change in subcellular localization.

MIER2 and 3 were sequenced by the NIH Mammalian Gene Collection Program [8], then named based on homology in their ELM2 and SANT domains to MIER1. While only one MIER2 isoform has been isolated, 5 MIER3 isoforms have been identified. Isoform 1 has been designated the canonical sequence and isoform 3 differs from this sequence by a single amino acid (aa) deletion (aa277) in the ELM2 domain. Isoform 2 contains a 5aa insertion near the N-terminus, while isoform 4 is missing the first 63aa. Isoform 5 is a truncated variant containing only the N-terminal 119aa and thus lacks both ELM2 and SANT domains.

Very little is known about MIER2 or MIER3 proteins and function. Both are predicted to be nuclear proteins [9, 10] and MIER3 has been identified as a candidate breast cancer susceptibility gene [11]. Several large-scale proteomic/interactome studies have identified MIER proteins in association with HDAC1 and/or HDAC2 [12–15]. Furthermore, such studies have demonstrated that MIER1, 2 & 3 are not components of the CoREST, NuRD, Sin3 or NCoR corepressor complexes, but rather form distinct HDAC-containing complexes.

While large-scale interactome analyses are very useful for providing information about the potential function of uncharacterized genes, it is important to validate any identified activities and/or properties. In this paper, we have begun to characterize MIER2 and MIER3 and compare them to MIER1α. We investigate their subcellular localization, their potential association with each other, their interaction with HDAC1 and 2, the activity of associated deacetylases and key residues for HDAC recruitment.

## Materials and Methods

### Cell lines and culture conditions

The MCF7 human breast adenocarcinoma cell line (HTB22™), Human Embryonic Kidney 293 (HEK293) cell line (CRL 1573™) and the HeLa cell line (CCL-2™) were obtained from the American Tissue Culture Collection (ATCC). All cell lines were cultured in DMEM (Thermo Fisher Scientific) containing 10% serum (7.5% calf serum (Thermo Fisher Scientific) plus 2.5% fetal bovine serum (Thermo Fisher Scientific) and 1mM sodium pyruvate (Thermo Fisher Scientific). Cells were grown a humidified 37°C incubator with 5% CO_2_.

### Plasmids and constructs

The human *mier1* gene structure, the sequence of its transcripts and the myc-tag vector containing full-length *mier1α* have been described in [2]. *Mier1*α (GenBank accession no. AY124187) was subcloned from pCS3+MT-*mier1*α into the EcoRI and XhoI sites of the pCMV-FLAG vector (Clontech Laboratories) to produce an N-terminal FLAG-tagged MIER1α protein.

*Mier2* cDNA (GenBank accession no. BC028203) in pCMV-SPORT6 was purchased from Dharmacon. An N-terminal myc-tagged construct was produced by subcloning the *mier2* coding region into the EcoRI and XbaI sites of the pCS4+MT vector [16] by PCR, using the following primers: 5’-GCG AAT TCA CCA TGG CGG AGG CCT CCT CGC-3’ (forward); 5’-GCT CTA GAT CAG CAG GTC ATC ACG TTA CAG-3’ (reverse). An N-terminal FLAG-tagged construct was produced by subcloning the *mier2* coding region into the EcoRI and HindIII sites of the pCMV-FLAG vector by PCR using the following primers: 5’-GCG AAT TCA CCA TGG CGG AGG CCT CCT CGC-3’ (forward); 5’-GCA AGC TTT CAG CAG GTC ATC ACG TTA CAG-3’ (reverse).

*Mier3* cDNA (GenBank accession no. NM_152622.3) in the pReceiver-M51 plasmid was purchased from GeneCopoeia. An N-terminal myc- or FLAG-tagged *Mier3* construct was produced by subcloning the coding region into the EcoRI and XhoI sites of the pCS4+MT vector or pCMV-FLAG vectors by PCR using the following primers: 5’-GCG AAT TCA CCA TGG CGG AGG CTT CTT TTG G-3’ (forward); 5’-GCC TCG AGT CAC TCA GAG TGT AGG GC-3’ (reverse).

*Mta1* cDNA in the pEnter vector was purchased from ViGene Biosciences. and subcloned by PCR into the EcoRI and XhoI sites of the pCS4+MT and pCMV-FLAG vectors using the following primers: 5’-GC GAA TTC ACC ATG GCC GCC AAC ATG TAC AGG-3’ (forward); 5’-GC CTC GAG CTA GTC CTC GAT GAC GAT GGG-3’ (reverse).

*Mier2* deletion constructs were produced by PCR using the following primers: (MIER2-∆1) 5’-GCG AAT TCA CCA TGG CGG AGG CCT CCT CGC-3’ (forward), 5’-GCT CTA GAT CAC ACA GAG CCC ATC TCG GAT C-3’ (reverse); (MIER2-∆2) 5’-GCG AAT TCA CCA TGT GGA GTG AAG AGG AGT GCA GG-3’ (forward), 5’-GCT CTA GAT CAG CAG GTC ATC ACG TTA CAG- 3’ (reverse); (MIER2-∆3) 5’-GCG AAT TCA CCA TGA AGA AGG AGA TCA TGG TGG GA-3’ (forward), 5’-GCT CTA GAT CAC TTC TTC CAC AGG TAG TA-3’ (reverse); (MIER2-∆4) 5’-GCG AAT TCA CCA TGA AGA AGG AGA TCA TGG TGG GA-3’ (forward), 5’-GCT CTA GAT CAA GCA CAG AGC CCA TCT CGG AT-3’ (reverse). PCR products were ligated into the EcoRI and XbaI sites of the pCS4+MT vector to produce N-terminal Myc-tagged proteins.

*Mier3* deletion constructs were produced by PCR using the following primers: (MIER3-∆1) 5’- GCG AAT TCA CCA TGG CGG AGG CTT CTT TTG G-3’ (forward), 5’- GCC TCG AGT CAT GCA GTC ATT CCT TG-3’ (reverse); (MIER3-∆2) 5’- GGG AAT TCA CCA TGT GGA CGG AAG AAG AAT GC-3’ (forward), 5’- GCC TCG AGT CAC TCA GAG TGT AGG GC- 3’ (reverse); (MIER3-∆3) 5’- GGG AAT TCA CCA TGA GGA AGG AAA TAA TG -3’ (forward), 5’- GCC TCG AGT CAT TTC TTC CAC ATA TA-3’ (reverse); (MIER3-∆4) 5’- GGG AAT TCA CCA TGA GGA AGG AAA TAA TG -3’ (forward), 5’- GCC TCG AGT CAT GCA GTC ATT CCT TG-3’ (reverse). PCR products were ligated into the EcoRI and XhoI sites of the pCS4+MT vector to produce N-terminal Myc-tagged proteins.

Myc-tagged MIER2 containing a point mutation ^228^W→A in the ELM2 domain was produced using the QuikChange site-directed mutagenesis kit (Stratagene) and the following primers: 5’-AGA CCA GCT GCT CGC GGA CCC CAG CGT C-3’ (forward); 5’- GAC GCT GGG GTC CGC GAG CAG CTG GTC T-3’ (reverse). Myc-tagged MIER3 containing a point mutation ^208^W→A in the ELM2 domain was produced using the QuikChange site-directed mutagenesis kit (Stratagene) and the following primers: 5’-AGT ATA TGA AAA CGA AGA CCA GTT ACT TGC GTG TCC TGA TGT GGT-3’ (forward); 5’-ACC ACA TCA GGA CAC GCA AGT AAC TGG TCT TCG TTT TCA TAT ACT-3’ (reverse).

All plasmids were prepared using the NucleoBond Endotoxin-free Maxi Plasmid kit (Clontech Laboratories), according to the manufacturer’s instructions. The sequences/mutations were confirmed by automated dideoxynucleotide sequencing of both strands (DNA Sequencing Facility, The Centre for Applied Genomics, The Hospital for Sick Children, Toronto, Canada).

### Antibodies

The 9E10 anti-myc tag mouse monoclonal antibody was prepared as described in Blackmore *et al*. [17]. The anti-HDAC1 antibodies (H-51, polyclonal) and the anti-HDAC2 antibody (H-54, polyclonal) were purchased from Santa Cruz Biotechnology. The monoclonal anti-FLAG M2 antibody (F1801) was purchased from Sigma-Aldrich. For confocal analysis, Alexa Fluor-488 labeled donkey anti-mouse (715-546-151) was purchased from Jackson ImmunoResearch Laboratories. HRP-labeled sheep anti-mouse (45000679) and donkey anti-rabbit (45000682) antibodies were purchased from GE Healthcare. HRP-label goat-anti mouse light chain-specific (115-035-174) was purchased from Jackson ImmunoResearch Laboratories.

### Transient transfection

MCF7 cells were transfected by electroporation using the Neon® electroporation device (Thermo Fisher Scientific) and the following settings: 1000V, 30ms, 2 pulses. 3x10^5^ MCF7 cells were mixed with 0.5μg myc-tagged plasmid and loaded into a 10μl tip for electroporation. HEK293 cells and HeLa cells (3x10^5^ cells) were transfected with 0.5μg plasmid using Mirus TransIT-LT1 transfection reagent (Medicorp) in a 3:1 ratio of reagent:DNA (v/w), according to the manufacturers’ protocol. After transfection, cells were plated at a density of 4x10^4^/well in Falcon 8-well culture slides (BD BioSciences) for confocal analysis or at 5x10^5^ /well in a 6-well dish for co-immunoprecipitation (co-IP) and Western analysis. Transfected cells were cultured for a total of 48hr before processing for analysis.

### Immunofluorescence, confocal microscopy and analysis

Transfected cells were fixed for 10 min with 4% paraformaldehyde/PBS and permeabilized with 0.1% Triton X-100/PBS for 15 min. Non-specific sites were blocked with 5% donkey serum in PBS for 1hr before overnight incubation with 9E10 antibody (1:200 dilution) at 4°C. Subsequently, the cells were incubated with Alexa Fluor-488 labeled donkey anti-mouse secondary antibody (1:400) for 1hr at RT. Nuclei were counterstained with 2.5μg/ml 4′, 6-diamidino-2-phenylindole (DAPI; Sigma-Aldrich) for 1hr. All slides were mounted in 10% glycerol/PBS. Cells were examined under an Olympus FluoView FV1000 confocal microscope. Fluorescence images were obtained by sequential z-stage scanning in two channels (DAPI and Alexa Fluor-488); z-stacks were compiled into individual images.

Quantitative analysis of confocal z-stacks was performed using Image J software v1.50g [18], as described in [3]. Briefly, cell outlines from compiled z-stacks were traced and the sum of the pixel values within the outlines in the 488 channel was determined. After subtracting the background, this value was used as the corrected whole cell MIER fluorescence. The sum of the pixel values for nuclei was determined in the same way and used as corrected nuclear MIER fluorescence. The nuclear value was subtracted from the whole cell value to obtain cytoplasmic MIER fluorescence. For each construct, the average nuclear and cytoplasmic values from 30–40 cells was calculated. Statistical analysis was performed with GraphPad software, using a two-sided Fisher’s exact test.

### Co-immunoprecipitation (co-IP) and western blot analysis

Transfected cells were washed once with 1xPBS and lysed on ice for 30 min in 1xIP buffer (1% Triton X-100, 150 mM NaCl, 20 mM Tris-Cl pH7.5, 1 mM EDTA, 0.02% Sodium Azide, 1 mM PMSF, 1% protease inhibitor cocktail). Cell lysates were passed several times through a 26-gauge needle then centrifuged at 12,000xg for 10 min at 4^°^C. The supernatants were incubated overnight at 4°C with 9E10 or anti-FLAG antibody (1:100 dilution). 50μl of 50% slurry of either Protein A-agarose beads (Pierce Biotechnology) or Protein G-agarose beads (EMD Millipore) was added to each sample and incubated for 1hr at 4°C. After incubation, the beads were washed 3 times with ice-cold 1xIP buffer and once with 150mM NaCl; bound proteins were solubilized in 30μl of 1.5x SDS sample buffer (50mM Tris-Cl pH6.8, 2% SDS, 5% β-mercaptoethanol, 10% glycerol, 0.1% bromophenol blue) and analyzed by SDS-PAGE-Western. Expression levels were determined by Western, using extracts of cells solubilized in SDS sample buffer.

Western blot analysis was performed as in [19] using 7.5% SDS-PAGE gels. Transfers were performed using 0.2μm PVDF membranes (Trans-Blot TurboTM Transfer Pack; Bio-Rad Laboratories) and the Trans-Blot Turbo^TM^ system (Bio-Rad Laboratories) set at 2.5A, 25V for 10 min. Membranes were stained using a 1:2000 dilution of primary antibody, 1:5000 HRP-labeled secondary antibody and Clarity^TM^ ECL Western Blotting Substrate (Bio-Rad Laboratories).

Densitometric quantitation of the HDAC1 and 2 bands co-immunoprecipitating with MIER1, 2 and 3 was performed using Image J v1.50g. HDAC and MIER band intensity in each immunoprecipitate was measured and the background subtracted. To correct for variations in MIER expression between experiments, the MIER1α expression level was used to normalize MIER2 and MIER3 values. The ratio of HDAC:MIER was determined for each immunoprecipitate and the average ratio + S.D. was calculated.

### [^3^H]-acetate labeling of histones

Histones were labeled as in [4]. Briefly, HeLa cells were grown to a density of 1x10^6^ cells in a 100mm dish. The culture medium was removed and 3 mls of fresh DMEM containing 10mM sodium butyrate and 0.5mCi/ml ^3^H-sodium acetate was added. Cells were incubated in humidified 37^°^C incubator with 5% CO_2_ for 1hr after which the medium was removed and the cells were washed twice with PBS. Cells were homogenized in 1ml of ice-cold lysis buffer (50 mM Tris-HCl pH 8, 150 mM NaCl, 1% Triton X-100, 10 mM MgCl2, 10% glycerol, 1X protease inhibitors); the nuclei were collected by centrifugation at 1,000xg for 10 min and washed three times with lysis buffer and once with 10mM Tris-HCl pH 7.4, 13mM EDTA, pH 8.0. The pellet was resuspended in 0.1 ml ice cold deionized H_2_O (dH_2_O) and concentrated H_2_SO_4_ was added to a final concentration of 0.2M. The suspension was incubated on ice for 1hr and centrifuged at 15,000 g for 5 min. 1 ml of acetone added to the supernatant and the sample incubated overnight at -20°C. The pellet containing the histones was collected by centrifugation at 15,000xg for 5 min and air-dried. The histones were resuspended in 50μl dH_2_O, the protein quantified using a Qubit protein assay kit (Thermo Fisher Scientific) and the ^3^H incorporated determined by liquid scintillation counting.

### Histone deacetylase assays

For each sample, 1x10^6^ cells were transfected with the appropriate construct and 48hr later, subjected to immunoprecipitation using the relevant antibody. For experiments measuring the effect of Ins(1,4,5,6)P4, Ins(1,4,5,6)P4 (Cayman Chemical) was added to a final concentration of 12.5μM δυρινγ ιμμuνοπρεχιπιτατιον; immunoprecipitates were collected on either Protein A or Protein G beads. HDAC assays consisted of immunoprecipitates, HDAC buffer (10 mM Tris-HCl pH 8.0, 150 mM NaCl, 1mM MgCl_2_), 5,000 cpm labeled histones, with or without 5μM trichostatin A (TSA; Sigma-Aldrich), in a final volume of 200μl. Samples were incubated at 37°C for 2hr on a half rotation, after which the reaction was terminated by the addition of 50μl Stop buffer (1M HCl/160mM acetic acid for labeled histones). Released ^3^H-acetate was extracted into 600μl ethyl acetate (Sigma-Aldrich) and the radioactivity determined using a liquid scintillation spectrometer (Beckman LS6500). Statistical analysis was performed using one-way ANOVA with post-hoc Tukey HSD.

## Results and Discussion

### MIER proteins display distinct regions of high homology

**Fig 1:**
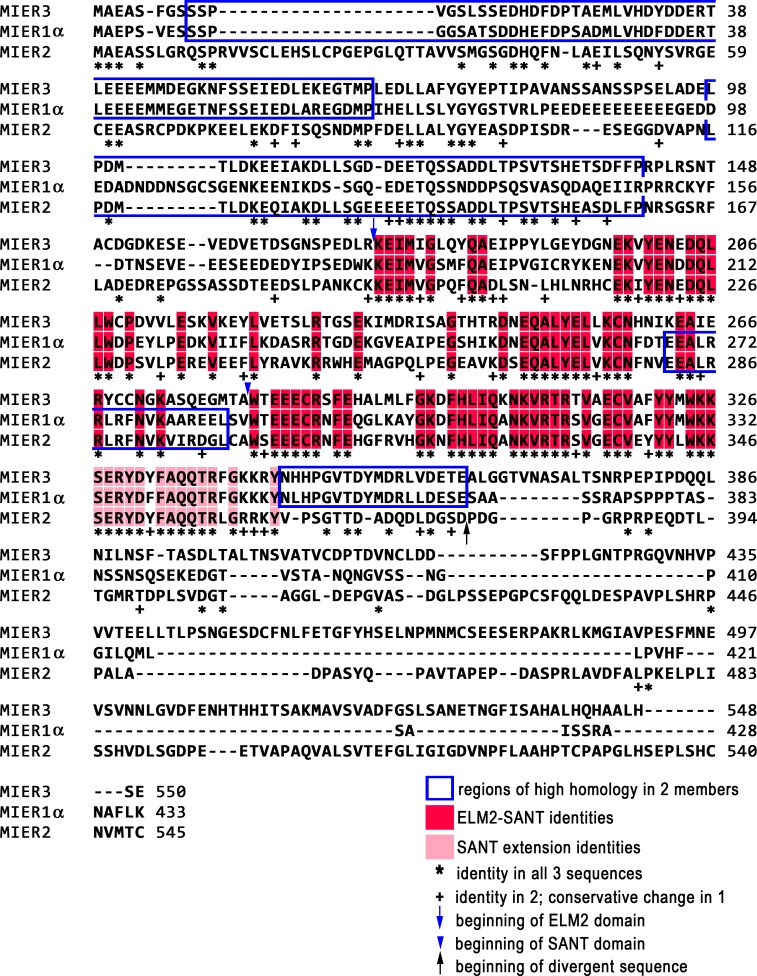
Alignment MIER1α, MIER2 and MIER3 protein sequences. The MIER1α, MIER2 and MIER3 isoform 1 protein sequences were aligned using MSAProbs [20]; gaps introduced by the alignment program are indicated by dashes and aa numbers are listed on the right. Identities in all 3 proteins are indicated by an ‘*’ and in the ELM2 and SANT domains, are also colored red. The beginning of the ELM2 and the SANT domain are indicated by a blue arrow and blue arrowhead, respectively. Identities in the region of high homology immediately downstream of the SANT domain (SANT extension) are colored pink. Identities in 2 of the 3 proteins are indicated by a ‘+’ sign. Regions of high homology (>70% identity) between 2 of the protein sequences are indicated by a blue outline. The beginning of the highly divergent C-terminal sequence is indicated by a black arrow.

Using the MSAProbs program [20], sequence alignment was performed using the α isoform of MIER1, the only isoform for MIER2 and the canonical isoform of MIER3, isoform 1. Alignment results revealed that both MIER2 and 3 display higher homology in the ELM2-SANT region to MIER1 (63% & 60% identity, respectively) than they do to each other (52% identity) (Table 1). The SANT domains of the three MIER proteins show a greater degree of conservation than do the ELM2 domains (70–82% identity vs 46–59%) (Table 1). Moreover, the 18aa immediately downstream of the SANT domain (SANT extension) are also highly conserved in all 3 proteins (72% identity, Table 1, Fig 1). This SANT extension was previously identified as a region that is 100% conserved in MIER1 from several different species, including mouse, rat, cow, chicken and *Xenopus* [21], suggesting that this region has an important functional role.

**Table 1:** Homology in various regions between MIER family members.

**REGION**	**MIER1α-MIER2**	**MIER1α-MIER3**	**MIER2-MIER3**
	% identity	% identity	% identity
**Overall**	42	50	36
**amino acid 9–65**	19	**74**	26
**amino acid 98–149**	35	33	**87**
**ELM2 (180–283)**	59	54	46
**ELM2 end (268–285)**	**78**	39	33
**SANT (288–332)**	80	82	70
**ELM-SANT (180–332)**	63	60	52
**SANT extension (333–350)**	78	89	83
**amino acid 351–368**	37	**84**	11

Apart from the ELM2 and SANT domains, very few functional domains have been characterized in the MIER proteins. Thus, comparison of the protein sequences for additional highly conserved regions might aid in the identification of putative functional motifs to investigate, while also enabling recognition of domains unique to individual family members. Sequence analysis revealed several regions of high homology between pairs of family members (blue outlines in Fig 1; bold in Table 1). MIER3 has two regions of high homology to MIER1; the first, located at the N-terminus (aa 9–65; numbers refer to positions in the MIER1 aa sequence) and containing the first two acid-rich regions [1], is 74% identical to MIER1 (Table 1; Fig 1), while MIER2 shows only 19% and 26% identity in this sequence to MIER1 and MIER3, respectively (Table 1). The second consists of a short region (aa 351–368) located immediately downstream of the SANT extension that is 84% identical to MIER1; this region in MIER2 shows only 37% and 11% identity to MIER1 and MIER3, respectively (Fig 1; Table 1). MIER2, on the other hand, contains one region of high homology (78%) to MIER1, located at the end of the ELM2 domain (aa268-285) (Fig 1; Table 1). This sequence contains one of two previously identified ALXXL motifs that are highly conserved in a number of ELM2 containing proteins [4]. MIER3 shows only 39% and 33% identity to MIER1 and MIER2, respectively and it lacks the second ALXXL motif. MIER2 and MIER3 share a specific region of high homology, located upstream of the ELM2 domain (aa98-149; Fig 1). This sequence is 87% identical in MIER2 and 3, but only 35% and 33%, respectively, to MIER1 (Table 1).

Interestingly, the C-termini of these 3 proteins are highly divergent, with only sporadic identities amongst them (Fig 1). The two major MIER1 isoforms, MIER1α and MIER1β, have distinct C-terminal ends, therefore we compared the β isoform C-terminus to determine if MIER2 and 3 are closer in homology to the β isoform. Alignment analysis revealed that the β C-terminus also shows only sporadic identities (S1 Fig), suggesting that member-specific functions might be accomplished through their unique C-termini.

### MIER2 and MIER3 are localized in the nucleus

**Fig 2:**
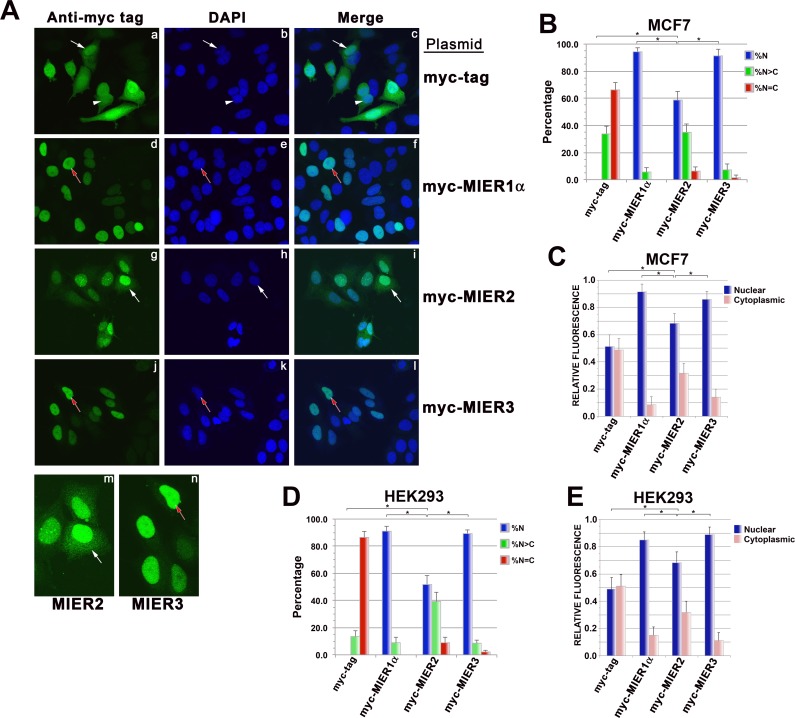
Confocal analysis of MIER2 and MIER3 subcellular localization. (A-C) MCF7 cells were transfected with a plasmid encoding myc tag alone or myc-tagged -MIER1α, -MIER2 or -MIER3 and localization was analyzed by confocal microscopy using DAPI (panels b, e, h, k) and the 9E10 myc tag antibody (AlexaFluor 488) (panels a, d, g, j, m, n). The merged DAPI and Alexafluor 488 channels are shown in panels c, f, i &l. (A) Illustrative examples of cells showing whole cell localization of the myc-tag (panels a-c), nuclear localization of MIER1α (panels d-f) and MIER3 (panels j-l) and mainly nuclear localization MIER2 (panels g-i). Panels m & n show and enlargement of the cells indicated by arrows in panels g & j; the brightness was increased in these panels to better illustrate the cyotplasmic localization of MIER2 (m), compared to exclusively nuclear localization of MIER3 (n). For all panels, white arrows indicate cells that display whole cell staining, with nuclear staining more intense than cytoplasmic (N>C); white arrowheads indicate cells showing whole cell staining with nuclear staining equal in intensity to cytoplasmic staining (N = C); red arrows with white outlines indicate cells with exclusively nuclear staining. (B) Histogram showing the percentage of cells, ± S.D., displaying each staining pattern: N, exclusively nuclear; N>C, nucleus more intensely stained than cytoplasm; N = C, nucleus and cytoplasm display equal staining intensity. Fields were selected at random and the staining pattern of all expressing cells in the field were scored visually from the compiled z-stacks. Only cells expressing myc-tagged proteins were included in the total counts and used to calculate percentages; 50–80 cells were scored for each construct. (C) Bar graph showing the intracellular distribution of each protein. Fields were selected at random and all cells expressing myc-tagged proteins in the field were analyzed, as described in the Materials and Methods. Pixel values for the nuclear and cytoplasmic compartments were measured in compiled confocal z-stacks using Image J v1.50 g. Plotted is the average value ± S.D. in each compartment, using measurements from 30–40 cells for each construct. (D-E) HEK293 cells were transfected with a plasmid encoding myc-tag alone or myc-tagged -MIER1α, -MIER2 or -MIER3 and localization was analyzed by confocal microscopy, as described for MCF7. (D) Histogram showing the percentage of cells ± S.D. in each of the categories described above. Localization was determined as described above for panel B; 50–80 cells were scored for each construct. (E) Bar graph showing the intracellular distribution of each protein, determined as described above in panel C. Plotted is the average value ± S.D. in each compartment, using measurements from 30–40 cells for each construct. For panels B-E, asterisks indicate values that are significantly different (p<0.05); there was no significant difference between the MIER1α and MIER3 values in any of the panels.

MIER2 and MIER3 are predicted to be nuclear proteins [9, 10] and we verified the subcellular localization of these proteins. MCF7 cells, transfected with plasmids encoding myc-tagged MIER1α, myc-tagged MIER2, myc-tagged MIER3 or myc-tag alone were examined by confocal microscopy (Fig 2). Confocal z-stacks were analyzed to determine the percentage of cells in which the fluorescence was exclusively nuclear or both nuclear & cytoplasmic; for the latter, cells were classified in two categories: 1) showing more intense staining in the nucleus than the cytoplasm (N>C) or 2) showing equal intensity (N = C). Quantitative measurements of fluorescence in the confocal z-stacks were also performed in order to determine the distribution in the nuclear and cytoplasmic compartments. As expected, the myc-tag alone was distributed throughout the cell (Fig 2A, panels a-c) with no cell showing exclusively nuclear staining (Fig 2B) and roughly equal amounts in the nuclear and cytoplasmic compartments (Fig 2C). Previously, we showed that MIER1α was localized in the nucleus of MCF7 cells [3, 16] and similar results were obtained here: 94% of cells displayed exclusively nuclear staining (Fig 2A, panels d-f; Fig 2B) and quantitative analysis showed that 92% of the protein is in the nuclear compartment (Fig 2C). MIER3 showed a similar pattern to MIER1α, with 91% of cells displaying exclusively nuclear staining (Fig 2A, panels j-l; Fig 2B) and 87% of the protein in the nuclear compartment (Fig 2C). MIER2, on the other hand, had a slightly different pattern. While the majority of cells (59%) displayed exclusively nuclear staining (Fig 2A, panels g-i; Fig 2B) and the majority of the protein (69%) is in the nuclear compartment (Fig 2C), there was a significant proportion of cells with staining in the cytoplasm as well (Fig 2A, panel m; 2B) and 31% of the protein was localized in this compartment (Fig 2C).

We verified the MIER2 and 3 subcellular localization pattern in another cell line: HEK293. As can be seen in Fig 2D–2E, the percentage of HEK293 cells displaying nuclear MIER2 or MIER3 (52% and 89%, respectively) was similar to that observed in MCF7, as was the proportion of MIER3 in the nuclear compartment (90%) and MIER2 in the cytoplasmic compartment (34%). The significance of MIER2 in the cytoplasm is unknown at the moment.

### MIER1, 2 and 3 exist in distinct complexes

**Fig 3:**
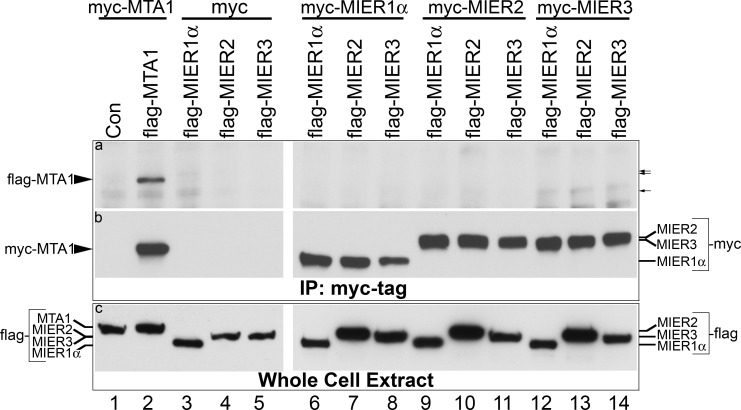
Co-immunoprecipitation analysis of flag-tagged with myc-tagged MIER proteins. HEK293 cells were transfected with a plasmid encoding myc-tagged MTA1 (lanes 1–2), myc tag alone (lanes 3–5), myc-tagged MIER1α (lanes 6–8), myc-tagged MIER2 (lanes 9–11) or myc-tagged MIER3 (lanes 12–14) along with either flag-tag alone (lane 1), flag-tagged MTA1 (lane 2), flag-tagged MIER1 (lanes 3, 6, 9 & 12), flag-tagged MIER2 (lanes 4, 7, 10 & 13) or flag-tagged MIER3 (lanes 5, 8, 11 & 14). Extracts were either subjected to immunoprecipitation with the 9E10 anti-myc tag antibody or directly loaded onto the gel. The immunoprecipitates (panel a) were analyzed by Western using a flag-tag antibody. The arrows to the right of panel a indicate the positions where flag-tagged -MIER2, -MIER3 and -MIER1α are expected to run (upper to lower arrow, respectively). The blot was stripped and restained with the 9E10 antibody (panel b) to verify the presence of myc-tagged MIER or MTA1 protein in each immunoprecipitate. Expression of the flag-tagged MIER proteins was verified using whole cell lysates from parallel samples and Western analysis with an anti-flag-tag antibody (panel c).

MIER1α bears a lot of similarity to MTA1, both in sequence and function. Both proteins contain contiguous ELM2-SANT domains, recruit HDACs, interact with ERα to repress its activity and both function generally as corepressors [4, 6, 22–24]. The MTA family of proteins is encoded by three genes, *mta1-3*, with MTA1 being the best characterized [25]. MTA1, 2 & 3 are components of the NuRD corepressor complexes [22] and stoichiometric analyses have demonstrated that the 3 proteins generally exist in distinct NuRD complexes [26], however all three have been detected in the MBD3/NuRD complex [27, 28]. Moreover, MTA1 has been shown to exist as a homodimer through interaction mediated by its ELM2 domain [25]. Therefore we investigated whether any of the MIER proteins co-exist in the same complex by performing co-immunoprecipitations of MIER complexes from cells expressing myc- and flag-tagged MIER1, 2 or 3 in various combinations. As shown in panel ‘a’ of Fig 3, when myc-tagged MIER1α, 2 or 3 is co-expressed with flag-tagged MIER1α, 2 or 3 and complexes are immunoprecipitated using a myc-tag antibody, no co-precipitating flag-tagged MIER protein was detected (lanes 6–14). As a positive control, flag-tagged MTA1 was shown to co-immunoprecipitate with myc-tagged MTA1 (Fig 3, panel a, lanes 1–2). We also confirmed the presence of the myc-tagged MIER protein in each co-IP and that each flag-tagged protein was expressed. This was done by re-staining the blot in panel ‘a’ using the myc-tag antibody and by performing Western analysis on whole cell extracts of parallel samples, using an anti-flag antibody. As can be seen in Fig 3, panel b, lanes 6–14, each myc-tagged MIER protein was present and levels were similar to that of the positive control, myc-tagged MTA1 (Fig 3, panel b, lane 2). Likewise, each flag-tagged protein was expressed (panel c, lanes 6–14) at a level at least as high as that of the positive control, flag-tagged MTA1 (panel c, lane 2). These results not only demonstrate that MIER proteins do not co-exist in the same complex, but also indicate that each complex contains only one molecule of MIER protein.

### MIER2 and 3 are not as effective as MIER1α at recruiting HDACs

**Fig 4:**
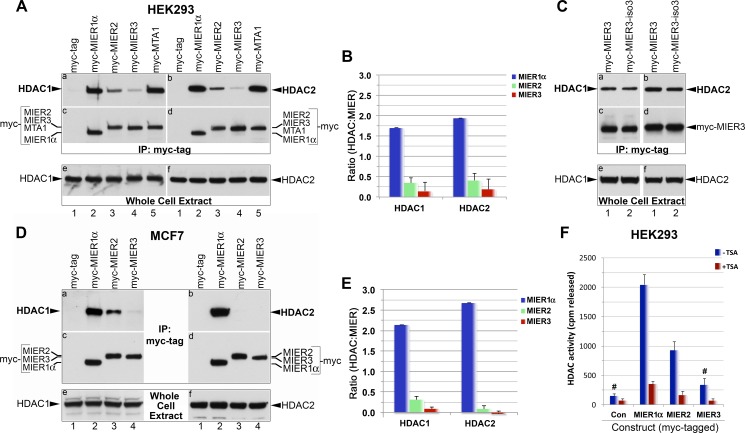
Co-immunoprecipitation of HDAC1 and 2 with MIER proteins. (A) HEK293 cells were transfected with a plasmid encoding myc tag alone (lane 1) or myc-tagged -MIER1α (lane 2), -MIER2 (lane 3), -MIER3 (lane 4) or -MTA1 (lane 5). Extracts were either loaded directly on the gel (panels e-f) or subjected to immunoprecipitation with the 9E10 anti-myc tag antibody (panels a-d). The immunoprecipitates were analyzed by Western using either anti-HDAC1 (panel a) or anti-HDAC2 (panel b). The blots in panels a & b were stripped and restained using the 9E10 anti-myc tag antibody (panels c-d) to verify levels of MIER protein in each immunoprecipitate. Whole cell extracts were analyzed by Western using anti-HDAC1 (panel e) or anti-HDAC2 (panel f) to verify equivalent HDAC levels in each sample. This experiment was performed 4 times. (B) Quantitation of the HDAC and MIER band intensity in the HEK293 immunoprecipitates was performed by densitometry using Image J 1.50g, as described in the Materials and Methods. Plotted is the average HDAC:MIER ratio ± S.D. for the 4 experiments from (A) and 3 experiments from Fig 5A (minus IP4 values only). (C) HEK293 cells were transfected with a plasmid encoding myc-tagged -isoform 1 (lane 1) or -isoform 3 (lane 2) of MIER3. The experiment was performed twice and Western analysis was completed as in (A). Note that a longer exposure than in (A) is shown here, in order to enhance visualization of the MIER3 associated HDAC bands. (D) MCF7 cells were transfected with a plasmid encoding myc-tag alone (lane 1) or myc-tagged -MIER1α (lane 2), -MIER2 (lane 3) or -MIER3 (lane 4). Western analysis was performed as in (A). (E) Quantitation of the HDAC and MIER band intensities in the MCF7 immunoprecipitates was performed as described in (B). Plotted is the average HDAC:MIER ratio ± S.D. for 4 experiments. (F) HEK293 cells were transfected with a plasmid encoding myc-tag alone or myc-tagged -MIER1α, -MIER2 or -MIER3. Extracts were subjected to immunoprecipitation with the 9E10 anti-myc tag antibody. Immunoprecipitates were assayed for histone deacetylase activity using [^3^H]-labeled histones as described in the Methods and Materials. HDAC assays, with or without TSA, were performed on duplicate samples and plotted is the average of three experiments ± S.D. Statistical analysis, using one-way ANOVA with post-hoc Tukey HSD, revealed all -TSA values were significantly different each other (p<<0.05), except for MIER3 when compared to Con; # indicates values that are not significantly different (p>0.05).

**Fig 5:**
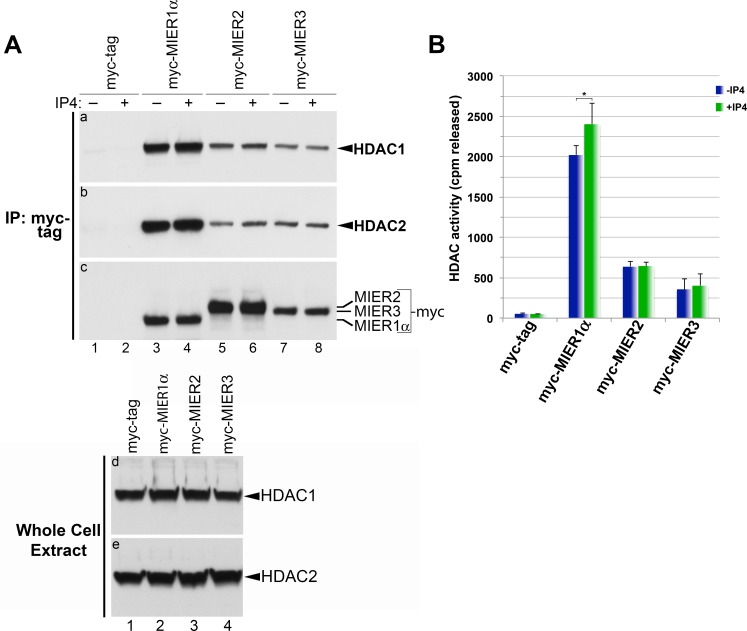
HDAC activity in the presence of Ins(1,4,5,6)P4. HEK293 cells were transfected with a plasmid encoding myc tag alone or myc-tagged -MIER1α, -MIER2 or -MIER3. Extracts were subjected to immunoprecipitation with the 9E10 anti-myc tag antibody, either in the absence (A, panels a-c, lanes 1,3,5,7; B) or presence (A, panels a-c, lanes 2,4,6,8; B) of Ins(1,4,5,6)P4. Immunoprecipitates were either analyzed by Western for associated HDAC1 and 2 (A) or assayed for histone deacetylase activity using [^3^H]-labeled histones (B). (A) Western blot analysis of immunoprecipitates using anti-HDAC1 (panels a); the blot in panel a was stripped and restained using anti-HDAC2 (panel b) and finally using the 9E10 anti-myc tag antibody (panel c). Whole cell extracts were analyzed by Western, using anti-HDAC1 (panel e) or anti-HDAC2 (panel f) to verify equivalent HDAC levels in each sample. (B) Immunoprecipitates, incubated with or without Ins(1,4,5,6)P4, were assayed for histone deacetylase activity, as described in the Methods and Materials. Assays were performed on duplicate samples and plotted is the average of three experiments ± S.D. Statistical analysis of the effect of Ins(1,4,5,6)P4 was performed using a standard t-test; asterisks indicate values that are significantly different when Ins(1,4,5,6)P4 is added (p< 0.05).

Previously, we demonstrated that MIER1 interacts with HDAC1&2 [4, 16]. More recently, Joshi *et al*. [13] employed a mass spectrometry-proteomic approach to characterize protein interactions for all 11 HDACs and in addition to MIER1, they detected MIER2 and 3 in HDAC1 and HDAC2 complexes. By contrast, proteomic target profiling of HDAC inhibitors by Bantscheff *et al*. [12] identified MIER2 and 3 with HDAC2, but not with HDAC1. To investigate and validate these MIER-HDAC interactions, we performed co-immunoprecipitation analysis using extracts from HEK293 cells expressing myc-tagged MIER1α, MIER2 or MIER3. Myc-tagged-MTA1 was also included as a positive control. As has been demonstrated previously [16, 29], both HDAC1 and 2 are present in MIER1α and MTA1 immunoprecipitates (Fig 4A, panels a-b, lanes 2 & 5). Likewise, both HDACs co-precipitated with MIER2 (Fig 4A, panels a-b, lane 3), albeit at lower levels than that seen with MIER1α. HDAC1 and 2 were also present in MIER3 immunoprecipitates (Fig 4A, panels a-b, lane 4), however levels varied between experiments and ranged from being barely detectable above the control (Fig 4A, panels a-b, lane 1) to the levels shown in Fig 5A. Therefore, to provide a more accurate representation of the level of interaction, we quantified the bands by densitometry using blots from all experiments for these two Figs and plotted the average ratio of HDAC:MIER in the immunoprecipitate. This analysis revealed that the level of HDAC 1 and 2 associated with the MIER2 complex was 20% of that in the MIER1α complex (Fig 4B). The MIER3 complex contained even lower levels of HDAC1 and 2, approximately 9% of that in MIER1α complex. The lower level of HDAC1/2 recovered with MIER2 & 3 was not due to a difference in MIER2 or 3 expression since re-staining of the blots in Fig 4A, panels a & b with an anti-myc tag antibody (Fig 4A, panels c-d) revealed expression levels similar to that of MIER1α (compare lanes 3 & 4 to lane 2 in Fig 4A, panels c-d). In addition, HDAC1 & 2 levels were comparable among the samples (Fig 4A, panels e-f).

Isoform 3 of MIER3 differs from isoform 1, used in the above experiments, by a single amino acid deletion (^277^E) in the ELM2 domain. Since this domain has been shown to be responsible for MIER1 interaction with HDACs, we investigated whether this deletion affects HDAC recruitment by MIER3. Co-immunoprecipitation analysis using extracts from HEK293 cells expressing myc-tagged isoform 1 or isoform 3 of MIER3 did not reveal any substantial difference in HDAC 1 or 2 levels (Fig 4C, panels a-b, compare lanes 1&2). Restaining of panels a-b for MIER3 showed equivalent expression levels (panels c-d); likewise, analysis of whole cell extracts revealed equivalent levels of HDAC1 and 2 expression in the samples (panels e-f). These results demonstrate that the ^277^E in the MIER3 ELM2 domain is not critical for HDAC recruitment.

Our data show that MIER2 and 3 are much less effective than MIER1α at recruiting HDAC1/2. To verify that is not a HEK293-specific result, we repeated this analysis using MCF7 cells. Interestingly, only HDAC1 co-immunoprecipitated with MIER2 in this cell line (Fig 4D, panels a-b, lane 3; Fig 4E) and no significant levels of HDAC1 or 2 was detected with MIER3 (Fig 4D, panels a-b, lane 4; Fig 4E). Given the variation in HDAC recruitment between these two cell lines, a third cell line, HeLa, was tested. The results obtained with HeLa cells were similar to that obtained with MCF7: only HDAC1 was associated with MIER2 and neither was detected with MIER3 (S2 Fig).

Our results with HEK293 are consistent with those reported by Joshi *et al*. [13] who used the CEM-T lymphoblast cell line, but are in contrast to those reported by Bantscheff *et al*. [12] who used the myelogenous leukemia cell line K562. In the former study, all 3 MIERs were identified in the HDAC1 and HDAC2 interactome, whereas in the latter study, MIER2 and 3 only interacted with HDAC2. Likewise, our results with MCF7 and HeLa differed from that obtained with HEK293 cells. These data serve to emphasize the cell line-dependent variability in the composition of MIER-HDAC complexes.

Next, we assessed the deacetylase activity of MIER-associated HDAC, using ^3^H-labelled core histones in combination with complexes immunoprecipitated from HEK293 cells expressing myc-tag alone or myc-tagged MIER1α, 2 or 3 proteins and then measuring the ^3^H-acetate released. As has been shown previously [4], MIER1α complexes contain HDAC activity that is inhibited by TSA (Fig 4F) and therefore MIER1α serves as a positive control, while the myc-tag alone provides the background control level. MIER2 immunoprecipitates contained significant levels of TSA-sensitive HDAC activity above background (Fig 4F), demonstrating that MIER2 complexes contain functional HDACs. Unlike MIER2, no significant HDAC activity above control levels was detected in MIER3 immunoprecipitates (p = 0.276; Fig 4F). The low level of HDAC1/2 recruitment by MIER3, even though MIER3 levels in the cell are high due to exogenous expression, and the fact that no MIER3-associated deacetylase activity could be detected, lead us to conclude that MIER3 is unlikely to function in HDAC recruitment under physiological conditions. An alternate explanation is that MIER3 requires another molecule, or co-factor, or specific environmental condition to activate its ability to interact with HDACs.

### Ins(1,4,5,6)P4 does not enhance HDAC activity or recruitment by MIER2 or 3

Millard *et al*. [29] reported that D-myo-inositol-1,4,5,6-tetrakisphosphate (Ins(1,4,5,6)P4) enhances the deacetylase activity of both HDAC3:SMRT and HDAC1-MTA1 complexes. Based on their results, these authors proposed that Ins(1,4,5,6)P4 activates all ELM2-SANT associated class I HDACs. We explored the possibility that Ins(1,4,5,6)P4 might increase HDAC activity and/or HDAC recruitment by MIERs. Extracts from HEK293 cells expressing myc-tagged MIER1, 2 or 3 were subjected to immunoprecipitation in the presence or absence of Ins(1,4,5,6)P4. Immunoprecipitates were analyzed by Western blot for the presence of HDAC1 and 2 proteins; parallel samples were tested for HDAC activity. No substantial difference in HDAC recruitment by any of the MIER proteins was detected when Ins(1,4,5,6)P4 was present (Fig 5A, panels a-b). A small, but statistically significant, increase in HDAC activity of MIER1 complexes was observed in the presence of Ins(1,4,5,6)P4 (Fig 5B), however no difference was detected in the deacetylase activity of either MIER2 or MIER3 complexes. The data presented here do not support the hypothesis that Ins(1,4,5,6)P4 is required for class 1 HDAC activation [29]. However, they do illustrate differences in the three members of the MIER family and presumably the complexes that they form.

### An intact ELM2 domain is required for recruitment of HDAC1 and HDAC2 by MIER2

**Fig 6:**
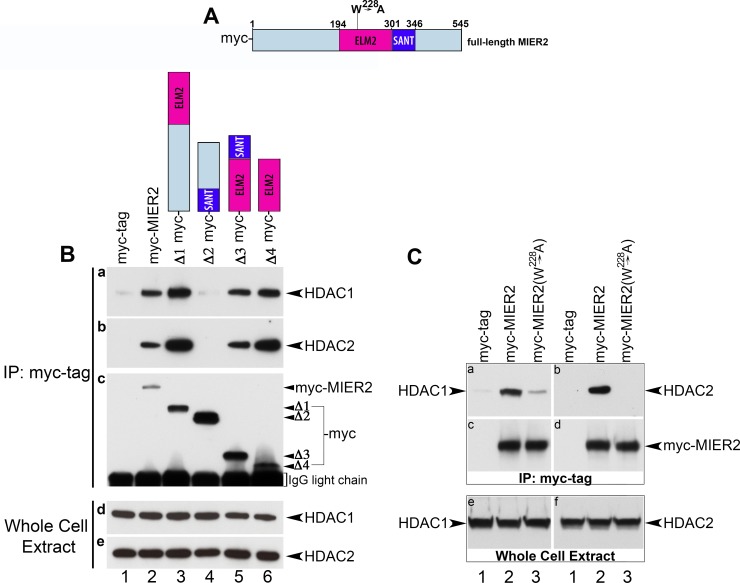
Interaction of HDAC1 and 2 with MIER2 deletion constructs. (A) Schematic showing a scaled representation of the MIER2 protein sequence, indicating the location of the ELM2 (dark pink) and SANT (dark blue) domains; the remaining sequence is colored light blue. The amino acid numbers for the beginning and end of the protein as well as beginning and end of ELM2 & SANT domains are indicated above the schematic. (B) HEK293 cells were transfected with a plasmid encoding myc tag alone (lane 1), myc-tagged full-length MIER2 (lane 2) or one of the following myc-tagged deletion constructs: Δ1 (aa1-301; lane 3), Δ2 (aa302-545; lane 4), Δ3 (aa194-346; lane 5), Δ4 (aa194-301; lane 6). Extracts were either loaded directly on the gel (panels d-e) or subjected to immunoprecipitation with the 9E10 anti-myc tag antibody. The immunoprecipitates were analyzed by Western, using either anti-HDAC1 (panel a) or anti-HDAC2 (panel b). The blots in panels a & b were stripped and restained using the 9E10 anti-myc tag antibody to verify the presence of the relevant MIER2 deletion construct in the immunoprecipitates; the restained blot from panel a is shown in panel c. The blots in panels d & e were stained with anti-HDAC1 or anti-HDAC2, respectively, to verify equivalent HDAC levels in the cell extracts. A schematic illustrating the MIER2 sequence included in the deletion construct used is shown above each lane. (C) HEK293 cells were transfected with a plasmid encoding myc tag alone (lane 1) or myc-tagged -wild-type MIER2 (lane 2) or -MIER2 containing a point mutation, ^228^W→A, in the ELM2 domain. Analysis was performed as in (B).

Previously, we demonstrated that the ELM2 of MIER1 is responsible for interaction with HDACs. Using deletion analysis with myc-tagged constructs, we investigated which region of the MIER2 sequence is required for recruitment of HDAC1 and 2. Deletion constructs consisted of: 1) an N-terminal half that included the ELM2 domain (Δ1); 2) a C-terminal half that included the SANT domain (Δ2); 3) ELM2 + SANT domains only (Δ3); 4) the ELM2 domain alone (Δ4). Expression of each construct was confirmed by Western blotting (Fig 6B, panel c), as was the expression level of HDAC1 and 2 in the cell extracts (Fig 6B, panels d-e).

Immunoprecipitates from extracts of HEK293 cells expressing full-length or one of the deletion constructs of MIER2 (Fig 6) were analyzed by Western blot for the presence of HDAC1 and HDAC2 (Fig 6B, panels a-b). Analysis of MIER2 revealed that HDAC1 & 2 only co-precipitated with constructs containing an ELM2 domain (Fig 6B, panels a-b, lanes 3–6) and that the ELM2 domain in isolation is sufficient for interaction (Fig 6B panels a-b, lane 6).

To further investigate the role of the ELM2 domain, we performed a mutational analysis. We had demonstrated previously that ^214^W in the ELM2 domain of MIER1 is a critical residue for recruitment of HDAC1/2 since mutation of this tryptophan to alanine resulted in loss of HDAC1/2 from the MIER1 complex. MIER2 contain a W in the equivalent position in its ELM2 domains (aa 228 in Fig 1), therefore we investigated the effect of mutating this residue on HDAC recruitment. Extracts from HEK293 cells expressing full-length wild-type or mutant MIER2 (Fig 6C) were subjected to immunoprecipitation and analyzed for the presence of HDAC1 and 2 by Western blot. Mutation of ^228^W to A abolished recruitment of HDAC1 and HDAC2 by MIER2 (Fig 6C, lane 3). These data demonstrate that MIER2 behaves similarly to MIER1 in recruitment of HDACs, with ^228^W in MIER2 being a critical residue.

What is evident from our data is that MIER family members do not dimerize or multimerize and thus form unique regulatory complexes. Our results show that only MIER1 and MIER2 recruit HDAC activity, but that recruitment levels and stoichiometry vary with cell type. What remains to be determined is the functional significance of this difference in HDAC recruitment and activity; this will require knowledge of the epigenetic and gene targets of MIER1, 2 and 3 complexes in order to elucidate their potentially distinct cellular functions.

## Conclusions

The data presented here constitute the first characterization of MIER2 and MIER3 proteins. Our results show that MIER2 and 3 are mainly localized in the nucleus. Only MIER2 can recruit HDAC1 and 2 activity, but this depends on cell type, and it does not do so as effectively as MIER1α. MIER proteins do not dimerize and Ins(1,4,5,6)P4 only enhances MIER1α associated HDAC activity. As with MIER1, there is a key tryptophan in the ELM2 domain of MIER2, ^228^W, that is required for HDAC recruitment.

## Supporting Information

S1 Fig Alignment MIER1β, MIER2 and MIER3 C-terminal sequences. The MIER1β, MIER2 and MIER3 protein sequences, beginning immediately after the SANT domain, were aligned using MSAProbs. Gaps introduced by the alignment program are indicated by dashes and aa numbers are listed on the right. Identities in all 3 proteins are indicated by an ‘*’ and in the SANT extension, are also colored pink. Identities in 2 of the 3 proteins is indicated by a ‘+’ sign. Regions of high homology (>70% identity) between 2 of the protein sequences is indicated by a blue outline. The beginning of the highly divergent C-terminal sequence is indicated by a black arrow.
(TIF)

S2 Fig Co-immunoprecipitation of HDAC 1 and 2 with MIER proteins in HeLa cells. Cells were transfected with a plasmid encoding myc tag alone (lane 1) or myc-tagged -MIER1α (lane 2), -MIER2 (lane 3) or -MIER3 (lane 4). Extracts were either loaded directly on the gel (panels e-f) or subjected to immunoprecipitation with the 9E10 anti-myc tag antibody. The immunoprecipitates analyzed by Western using either anti-HDAC1 (panel a) or anti-HDAC2 (panel b). The blots in panels a & b were stripped and restained using the 9E10 anti-myc tag antibody (panels c-d) to verify the levels of the relevant MIER protein in the immunoprecipitate. The blots in panels e & f were stained with anti-HDAC1 or anti-HDAC2, respectively, to verify equivalent HDAC levels in the cell extracts.
(TIF)
