# Supplementary figures and images for: Differential HDAC1 and 2 Recruitment by Members of the MIER Family
Source: PLoS One. 2017 Jan 3;12(1):e0169338. doi: 10.1371/journal.pone.0169338 (PMC5207708; doi:10.1371/journal.pone.0169338)

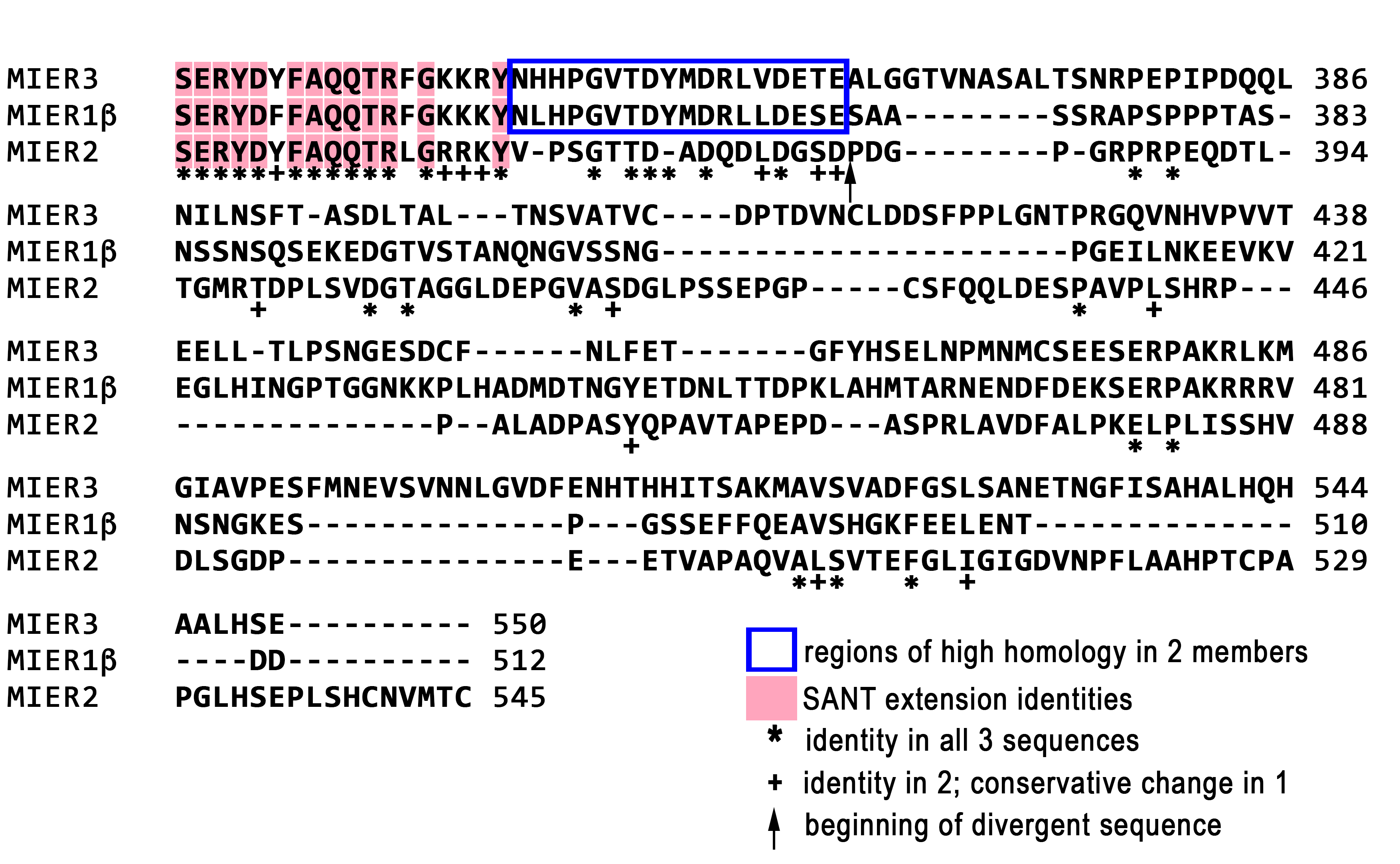

Supplement: S1 Fig — The MIER1β, MIER2 and MIER3 protein sequences, beginning immediately after the SANT domain, were aligned using MSAProbs. Gaps introduced by the alignment program are indicated by dashes and aa numbers are listed on the right. Identities in all 3 proteins are indicated by an ‘*’ and in the SANT extension, are also colored pink. Identities in 2 of the 3 proteins is indicated by a ‘+’ sign. Regions of high homology (>70% identity) between 2 of the protein sequences is indicated by a blue outline. The beginning of the highly divergent C-terminal sequence is indicated by a black arrow. (TIF) [file pone.0169338.s001.tif]

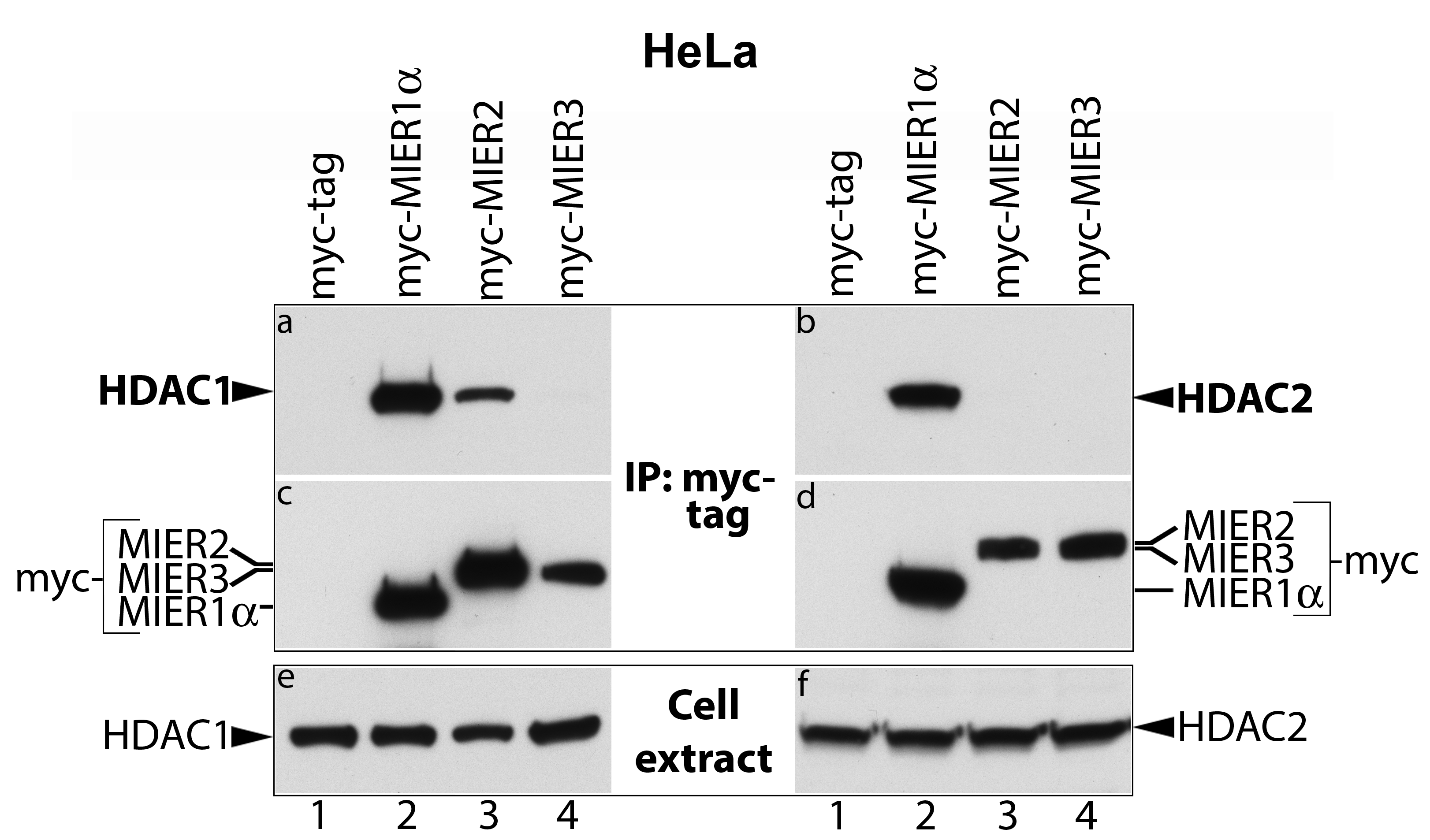

Supplement: S2 Fig — Cells were transfected with a plasmid encoding myc tag alone (lane 1) or myc-tagged -MIER1α (lane 2), -MIER2 (lane 3) or -MIER3 (lane 4). Extracts were either loaded directly on the gel (panels e-f) or subjected to immunoprecipitation with the 9E10 anti-myc tag antibody. The immunoprecipitates analyzed by Western using either anti-HDAC1 (panel a) or anti-HDAC2 (panel b). The blots in panels a & b were stripped and restained using the 9E10 anti-myc tag antibody (panels c-d) to verify the levels of the relevant MIER protein in the immunoprecipitate. The blots in panels e & f were stained with anti-HDAC1 or anti-HDAC2, respectively, to verify equivalent HDAC levels in the cell extracts. (TIF) [file pone.0169338.s002.tif]
